# Decomposing the rural–urban gap in hygienic material use during menstruation among adolescent women in India

**DOI:** 10.1038/s41598-023-49682-1

**Published:** 2023-12-16

**Authors:** Mahashweta Chakrabarty, Aditya Singh, Subhojit Let, Shivani Singh

**Affiliations:** 1 Department of Geography, Banaras Hindu University, Varanasi, Uttar Pradesh India; 2 Girl Innovation, Research, and Learning (GIRL) Center, Population Council, NY USA; 3 Independent Researcher, Lucknow, Uttar Pradesh India

**Keywords:** Health care, Risk factors

## Abstract

The use of hygienic materials (sanitary napkins, locally prepared napkins, tampons, and menstrual cups) during menstruation among adolescent women in India has improved over the years, yet a significant rural–urban gap in the usage persists at the national level. This study investigates how this rural-urabn gap varies across different states and union territories (UTs) of India and uses Fairlie decomposition to quantify the contribution of various factors to this gap. The study uses data on 114,805 adolescent women (aged 15–19 years) from the fifith round of National Family Health Survey (2019–21). The utilization of hygienic materials during menstruation among adolescent women in rural India stood at 43%, whereas in urban areas, it was 68%, indicating a significant 25 percentage point (pp) difference between the two. The rural–urban gap in the hygienic material use varied significantly across Indian states and UTs. The gap exceeded 20 pp in Madhya Pradesh (36 pp), Odisha (26 pp), Jammu and Kashmir (25 pp), Assam (25 pp), Uttar Pradesh (23 pp), Jharkhand (22 pp), Chhattisgarh (21 pp), and Rajasthan (21 pp). In contrast, the gap in Tamil Nadu, Himachal Pradesh, and Telangana was less than 10 pp. The decomposition analysis of the rural–urban gap (25 pp) revealed that the variables included in the anlaysis explained about 70% of the gap. The difference in the household wealth between rural and urban areas contributed about 69% of the explained gap. Other significant contributors to the explained gap were ‘transportation to health facility’ (5.6%), ‘mass-media exposure’ (4.9%), and ‘level of education (4.4%). The findings underscore the necessity for state-specific interventions aimed at vulnerable groups, particularly individuals from economically disadvantaged backgrounds, those with lower levels of education, and limited exposure to mass media, in order to reduce the existing rural–urban disparity in hygienic material use among adolescent women.

The onset of menstruation is one of the most profound changes in adolescent individuals, signifying the onset of their reproductive abilities^1–3^. Globally, there is a consensus that menstruating individuals should have access to hygienic materials, such as sanitary napkins, tampons, and menstrual cups to absorb or collect menstrual bloods^3–7^. These materials offer safety, comfort, and dignity during a woman's menstrual cycle^4^. However, evidence suggests that due to the high costs of hygienic materials, many women opt for a more economical but less hygienic alternatives that are washed and dried in unclean and poorly lit conditions^8–10^. Unfortunately, the use of these materials can lead to adverse health outcomes, including reproductive tract infections^9,11–13^.

Out of 1.8 billion menstruators worldwide, many face challenges in managing their menstrual health^7^. Among the most vulnerable groups are the adolescent women, who often grapple with restrictions on autonomy and control over their bodies^14,15^. Their limited financial independence and decision-making power within the household restricts their mobility and personal choices^14^. The situation is further exacerbated by the limited access to information on menstrual health and hygiene^10,16^. These constraints can lead them to experience feelings of depression, isolation, and frustration during menstruation^16–18^. This not only discourages them from discussing menstruation with friends and family, but also has a detrimental effect on their school attendance and reduces their engagement in community activities^16,19,20^.

Recognizing the significance of menstrual health and hygiene for the well-being and educational achievements of adolescent girls, both the Central and State Governments in India have implemented a range of menstrual health and hygiene schemes aligned with the Sustainable Development Goals, emphasizing inclusivity and equity with their motto, "no one left behind."^21,22^ Recent evidence indicates that, despite an improvement in the use of hygienic materials among adolescent women in India, significant disparities persist across various socioeconomic factors, including wealth, education, social group, religion, and geographic scales such as state, district^3,5,19,23–30^.

Notably, studies have also observed a substantial and persistent rural–urban disparity in the hygienic material use at the national level^5,27,31–35^. However, this aspect has not been thoroughly investigated. Given India's vast diversity and wide dimensions, it is imperative to explore how the rural–urban gap in hygienic material use among this demographic varies across the various states and union territories of the country. To bridge the rural–urban disparity in hygienic material use among adolescent women, it is crucial to gain a comprehensive understanding of the contributing factors. While prior studies in India have explored factors influencing menstrual product usage among adolescent and young women in rural and urban settings individually, none have specifically investigated the factors contributing to this rural–urban gap in hygienic material use, nor have they attempted to quantify the relative contribution of each of these factors to this gap^5,19,23,27,34,36^.

This study aims to investigate the variations in the rural–urban gap in hygienic material use among adolescent women across different geographical regions (states and union territories) and socioeconomic groups. Additionally, it seeks to decompose the rural–urban gap in hygienic material use among adolescent women in India and to quantify the contribution of various factors contributing to this gap.

## Methods

### Data source

The data for this study comes from the fifth round of the National Family Health Survey (NFHS-5), which was conducted during 2019–21. It is a multi-round, large-scale survey with a nationally representative sample of Indian households. The NFHS collects information on various demographic, socioeconomic, maternal and child health outcomes, morbidity and healthcare, reproductive health, and family planning issues^37^. The Ministry of Health and Family Welfare (MoHFW) of the Government of India approved the NFHS-5 and it was carried out by the International Institute of Population Sciences (IIPS), Mumbai, India. The NFHS-5 sample is a stratified two-stage sample. The study has been designed as a nationally representative cross-sectional study^37^. In the survey, a uniform multistage sampling technique has been adopted with separate sampling in urban and rural areas. Detailed information about the sampling employed in this survey can be obtained from the national report of NFHS-5^37^.

**Figure 1:**
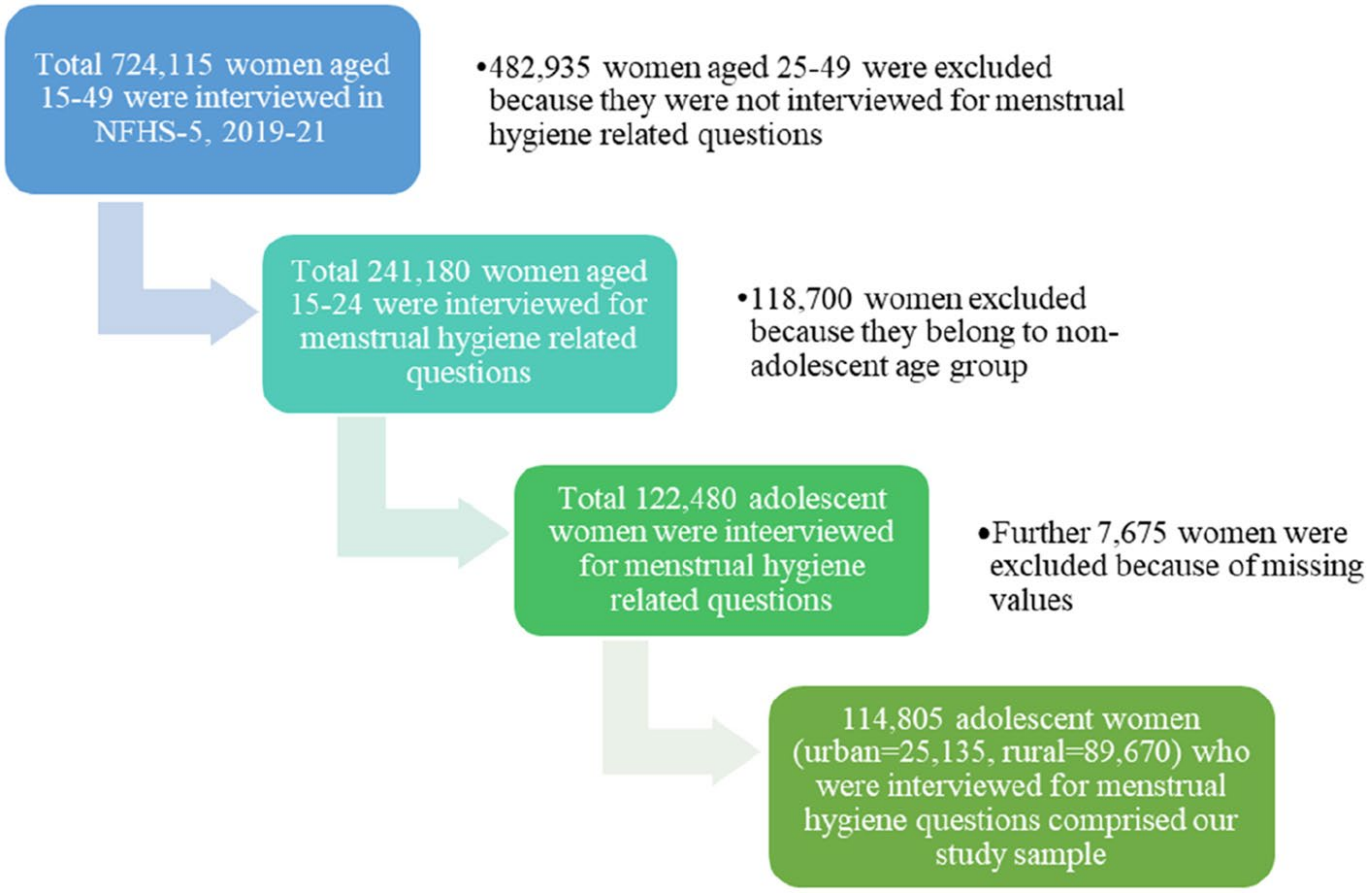
Procedure of sample size selection for the current study.

In NFHS-5, 724,115 women aged between 15 and 49 were interviewed from 636,699 households, covering 28 states, eight union territories, and 707 districts of India. For women, the response rate was 97% in the survey. Our study sample included 114,805 adolescent women (urban = 25,135, rural = 89,670) who were asked questions regarding their menstrual hygiene. The details of our sampling process are shown in Fig. 1.

### Outcome variable

During NFHS-5, women were asked about methods of protection they use during their menstrual cycle to avoid bloodstains from being evident. There were seven responses recorded to this question: (i) cloth, (ii) locally prepared napkins, (iii) sanitary napkins, (iv) tampons, (v) menstrual cups, (vi) nothing, and (vii) others^37^.

We created a binary outcome variable based on the recorded responses to the preceding question. During menstruation, the use of one or more of the following products: sanitary napkins, locally made napkins, tampons, and menstrual cups was classified as "exclusive use of hygienic materials" and coded as "1"^15,37^. The use of non-hygienic materials, such as cloths and ‘others’, or the use of both hygienic and non-hygienic materials combinedly, or not using any menstrual materials during menstruation was classified as "nonexclusive use hygienic materials" and coded as "0."^2,12,15,38^.

In this study, when we mention the "use of hygienic materials," we are specifically referring to the "exclusive use of hygienic materials". To maintain simplicity and clarity, we have opted to use the term "use of hygienic materials" consistently throughout.

### Independent variables

We examined an array of socioeconomic and demographic factors, including respondents' age at menarche, marital status, education, working status, religion, region of residence, social group, household wealth, exposure to mass media, interaction with community health care workers, bank account ownership, mobile phone ownership, the problems related with seeking medical help for oneself. The current literature on menstrual hygiene practices largely influenced the selection of these factors^2,11,12,24,39,40^.

It is worth noting that the variable "problem regarding getting medical help for self: getting money needed for treatment" serves as a proxy for affordability, as it reflects financial challenges in accessing medical assistance, which can extend to the affordability of hygienic menstrual materials. Similarly, we assessed accessibility by considering variables like "problem regarding getting medical help for self: distance of health facility" and "problem regarding getting medical help for self: transportation." These variables serve as proxies for the accessibility, indicating the difficulties individuals may encounter in reaching health facilities or obtaining necessary services. The details of the independent variables are given in Table 1.

**Table 1:** List of the independent variables used in this study.

Independent variables	Description
Age at menarche (in years)	Age at menarche refers to the age at which an individual experiences their first menstruation, marking the onset of their menstrual cycles. This variable has three categories: '12 years or younger' (1), 'between 13 and 15 years' (2), and '16 years or older' (3).
Marital status	The variable marital status has three categories: 'not-married' (0), 'married before 18 years' (1), 'married on or after 18 years' (2).
Level of education	Level of education is divided into four categories: 'no education' (0), 'primary' (1), 'secondary' (2), and 'higher' (3).
Religion	Religion has four categories– 'Hindu' (1); (b) 'Muslim' (2); 'Christian' (3); 'Others' (4).
Social group	The term "Social Group" refers to the official classification of castes and tribes used by the Central Government in India. This classification is instrumental in various administrative and policy-related contexts, aiding in the effective implementation of programs and policies targeted at specific social groups within the population. There are four types of social groups: Scheduled Castes (SC) (1), Scheduled Tribes (ST) (2), Other Backward Classes (OBC) (3), and Others (4).
Household wealth index	Wealth index, which has been developed and validated in many countries of the world to be a robust measure of household wealth and has been found to be consistent with measures of household income and expenditure^41^. It has been computed from the NFHS-5 survey data by combining household amenities and assets, and combined with weights derived from a principal component analysis procedure and then divided into quintiles, on the basis of which households were categorized from the poorest to the richest groups corresponding from the lowest to the highest quintiles. The wealth quintiles utilized in this study were labelled and coded as follows: 'poorest' (1), 'poorer' (2), 'middle' (3), 'richer' (4), and 'richest' (5).
Region of residence	Indian states and union territories (UTs) have been grouped into six ‘regions’. ‘North' (1) includes Jammu & Kashmir, Ladakh, Himachal Pradesh, Punjab, Rajasthan, Haryana, Uttarakhand, Chandigarh and Delhi; ‘Central' (2) includes the states of Uttar Pradesh, Madhya Pradesh and Chhattisgarh; ‘East' (3) includes the states of Bihar, Jharkhand, West Bengal and Odisha; ‘West' (4) includes the states of Gujarat, Maharashtra, Goa and UTs of Dadra & Nagar Haveli and Daman & Diu; ‘South' (5) includes the states of Kerala, Karnataka, Andhra Pradesh, Tamil Nadu and the UTs of Andaman & Nicobar Islands, Pondicherry and Lakshadweep); ‘North-east' (6) includes the states of Sikkim, Assam, Meghalaya, Manipur, Mizoram, Nagaland, Tripura, and Arunachal Pradesh. This classification has been used by the NFHS-5 report^42^.
Exposure to mass media	In the NFHS-5, women were asked three questions regarding their mass media exposure. These questions assessed the frequency of their exposure to various forms of media, including reading newspapers/magazines, watching television, and listening to the radio. The response options provided were: 'at least once a week,' 'less than once a week,' and 'not at all.' To simplify the analysis, these response options were converted into binary categories, specifically 'yes' or 'no,' indicating whether the respondent had exposure to each respective form of media. Subsequently, by combining the responses from these three binary variables, we constructed a new variable to measure the level of mass-media exposure for each respondent. This new variable comprised four categories: 'No exposure' (coded as 0): This category encompassed respondents who reported no exposure to any form of mass media 'Low exposure' (coded as 1): This category included respondents who reported exposure to only one type of mass media, such as either newspapers/magazines, television, or radio 'Medium exposure' (coded as 2): This category covered respondents who reported exposure to any two types of mass media 'High exposure' (coded as 3): This category encompassed respondents who reported exposure to all three types of mass media
Discussed menstrual hygiene with healthcare workers in past 3 months	Two questions were asked to the respondents in the NFHS-5, they are- i) in the last three months, if the respondent has met with any health worker- including an auxiliary nurse midwife (ANM), accredited social health activist (ASHA), *Anganwadi* worker (AW), also known as Integrated Child Development Services worker, multipurpose worker (MPW), or any other community health worker; and ii) if they have discussed about menstrual hygiene during the meeting. If respondent did not discuss menstrual hygiene with healthcare workers, then they are coded as 0, if discussed 1
Working status	Working status indicates the employment condition of the respondents. A dichotomous variable is formed: 'not working' (0) and 'working' (1)
Ownership of bank account	Whether a respondent owns a bank/savings account by herself – 'yes' (1); 'no' (0)
Ownership of mobile phone	Using this variable, we wanted to focus on whether the respondent owns a mobile phone personally. This variable has two categories; whether a respondent owns a mobile phone by herself – 'yes' (1); 'no' (0) It is noteworthy to emphasize that this variable is distinct from the mobile telephone ownership variable utilized in constructing the wealth index, which is a household-level variable. However, the household-level variable does not provide nuanced information regarding the ownership and usage patterns of individual women within that household. To address this limitation, our analysis includes a specific individual-level variable that focuses on whether the respondent, a woman in our study, owns and uses a mobile phone personally To ensure that the inclusion of this individual-level variable does not introduce any issues in our analysis, we conducted a thorough check for multicollinearity using Variance Inflation Factors (VIF) (detailed in Supplementary Table 3). VIF results confirmed the absence of multicollinearity among the independent variables
Problem regarding getting medical help for self: getting permission to go	This variable pertains to obtaining permission to seek medical help for oneself and is categorized based on the nature of the problem as follows: (a) no problem; (b) big problem; (c) not a big problem.
Problem regarding getting medical help for self: getting money needed for treatment	Women are queried about the extent to which getting money for medical treatment poses a challenge when seeking medical help for themselves. This variable has three categories: (a) no problem; (b) big problem; (c) not a big problem
Problem regarding distance of the health facility centre	This variable shows the extent to which the distance of primary health centre was a problem in getting medical help for self. This variable has three categories: (a) no problem; (b) big problem; (c) not a big problem
Problem of transportation to go for medical help for self	This variable shows nature of problem regarding transport that women face in order to get medical help for oneself and accordingly categorized as: (a) no problem; (b) big problem; (c) not a big problem

### Statistical analysis

Bivariate analysis has been used to examine the differences in the use of hygienic materials among adolescent women between rural and urban populations across various socioeconomic and biodemographic groups. To adjust for the complex survey design of NFHS-5, such as sampling weights, design effects, and clustering, we used "svyset" command in Stata 16, throughout the analysis^43^. Besides, we prepared state-level maps of the use of hygienic materials among adolescent women for the rural and urban population using QGIS 3.32.3^44^.

We used Fairlie decomposition method, a modified form of Blinder-Oaxaca (BO) decomposition, to identify and quantify the contribution of each predictor explaining the rural–urban gap in the hygienic materials use among adolescent women in India^45^. The BO decomposition is a method that orginiated in economics. It decomposes gap in mean outcome (e.g. wage) across two groups (e.g. men and women) into two parts: a) that is due to group differences in the levels of explanatory variables (also known as endowment effect or the explained gap) and a part that is due to differential magnitudes of regression coefficients (coefficient effect or the unexplained gap)^45^. This method is straightforward to use, requiring only coefficient estimates from linear regressions for the specific outcome and the sample means of the independent variables employed in these regressions^45^. However, a challenge emerges when the outcome is binary, such as use of hygienic materials, and the coefficients are derived from a logit or probit model. In such cases, these coefficient estimates cannot be directly applied in the standard BO decomposition equations. This problems is addressed by Fairlie decomposition which is an extension of the BO method^45^.

Since, the study uses a binary outcome variable, we used Fairlie decomposition method to achieve our objective of decomposing the rural–urban gap to identity factors contributing to the gap and quantify their relative contribution to the gap. For a detailed description of Fairlie decomposition method, please refer to Appendix-1. We have used the 'fairlie' command in Stata 16 to conduct the decompistion analysis^46^.

It is important to note that the independent variables were tested for possible multicollinearity by variance inflation factors (VIF) before entering them into the decomposition analysis. We found that multicollinearity (VIF < 2) was not a problem^47^ [for detailed VIF values, see supplementary Table 1, Additional file 1].

### Ethical approval and consent to participate

The present study used secondary data which is available in public domain. The dataset had no identifiable information of the survey participants. Therefore, no ethical approval was required for conducting this study.

## Results

Nearly half of the adolescent women in India (50%) reported use of hygienic materials during menstruation. However, rural adolescent women had a lower rate of hygienic material usage compared to their urban counterparts. Specifically, 43% of rural adolescent women reported use of hygienic materials for menstrual bloodstain prevention, whereas a higher proportion of 68% of their urban counterparts did the same.

Table 2 demonstrates a significant rural–urban gap in the use of hygienic materials during menstruation. This gap varies across different demographic and socioeconomic factors. For instance, among women with no education, the rural–urban gap in hygienic material use is approximately 20.9 percentage points (pp), with 35.6% of urban women compared to 14.7% of rural women utilizing hygienic materials. This discrepancy also holds true for women with primary, secondary, and higher education levels, with the gap ranging between 15 and 25 pp. The rural–urban gap in the use of hygienic materials during menstruation varied across the wealth quintiles (range: 6–11 pp).

**Table 2:** Use of hygienic materials among adolescent women by selected background characteristics in urban and rural India, NFHS-5 (2019–21).

Background characteristics	Urban (N = 25,135)		Rural (N = 89,670)		Gap (pp)
	Users#	% [95% CI]	Users#	% [95% CI]	
Age at menarche (in years) (χ2: 163.548, *p*: < 0.001)					
≤ 12	3599	71.3 [69.3,73.3]	6862	45.7 [44.4,47.0]	25.6
13–15	13,340	68.3 [66.9,69.6]	30,428	42.1 [41.5,42.8]	26.2
≥ 16	394	71.6 [65.9,76.6]	1189	49.3 [46.7,51.9]	22.3
Marital status (χ2: 201.494, *p*: < 0.001)					
Not married	16,209	69.6 [68.4,70.8]	32,696	42.9 [42.2,43.5]	26.8
Married before 18 years	533	56.5 [51.5,61.3]	3139	40.5 [38.7,42.2]	16.0
Married on or after 18 years	591	65.0 [60.6,69.3]	2643	47.1 [45.2,48.9]	18.0
Level of education (χ2: 5327.393, *p*: < 0.001)					
No education	195	35.6 [29.7,42.0]	697	14.7 [13.4,16.1]	20.9
Primary	321	36.2 [31.6,41.1]	1011	19.4 [17.9,20.9]	16.9
Secondary	14,656	69.9 [68.6,71.1]	33,309	44.9 [44.3,45.6]	25.0
Higher	2,161	79.4 [77.1,81.5]	3463	62.3 [60.5,64.1]	17.1
Religion (χ2: 840.686, *p*: < 0.001)					
Hindu	13,531	71.5 [70.2,72.7]	32,787	43.2 [42.5,43.8]	28.3
Muslim	2,782	56.1 [53.2,58.9]	3481	34.9 [33.2,36.7]	21.1
Christian	499	77.8 [72.8,82.2]	908	55.7 [52.4,59.1]	22.1
Others	521	86.6 [83.3,89.3]	1304	62.6 [59.9,65.2]	24.0
Social group (χ2: 1704.358, *p*: < 0.001)					
SC	3,695	67.7 [65.5,69.9]	9695	42.7 [41.6,43.9]	25.0
ST	703	64.3 [60.0,68.4]	4045	36.8 [35.4,38.2]	27.5
OBC	7613	65.6 [64.0,67.2]	17,312	41.6 [40.8,42.5]	24.0
Other	5322	76.2 [74.1,78.2]	7427	51.7 [50.3,53.1]	24.6
Household wealth index (χ2: 15,000.000, *p*: < 0.001)					
Poorest	316	34.0 [29.3,39.0]	6684	25.7 [24.8,26.5]	8.4
Poorer	938	44.4 [40.9,48.0]	9710	38.3 [37.3,39.2]	6.1
Middle	2587	57.9 [55.5,60.2]	10,401	51.8 [50.7,52.9]	6.1
Richer	5401	69.7 [67.9,71.3]	7799	61.6 [60.3,62.9]	8.1
Richest	8091	82.0 [80.7,83.3]	3886	70.7 [68.9,72.4]	11.3
Region of residence (χ2: 12,500.000, *p*: < 0.001)					
North	3313	80.5 [78.9,82.0]	7184	59.0 [57.6,60.4]	21.5
Central	3021	50.5 [48.2,52.8]	7434	25.0 [24.2,25.8]	25.5
East	2634	62.2 [58.8,65.5]	9675	39.9 [38.6,41.2]	22.3
West	3327	76.2 [73.1,79.1]	4973	56.9 [54.9,58.8]	19.4
Southern	4782	79.6 [77.6,81.4]	8377	68.0 [66.2,69.7]	11.6
North-east	256	60.2 [56.3,63.9]	836	34.1 [32.3,35.9]	26.1
Exposure to mass media (χ2: 7565.018, *p*: < 0.001)					
No exposure	836	44.7 [40.9,48.6]	5,388	25.2 [24.3,26.1]	19.5
Low exposure	7035	65.6 [63.9,67.2]	18,812	44.1 [43.3,44.9]	21.5
Medium exposure	8080	75.6 [74.2,77.0]	12,434	55.6 [54.5,56.6]	20.1
High exposure	1382	75.0 [71.7,77.9]	1845	57.1 [54.7,59.5]	17.8
Discussed menstrual hygiene with healthcare workers in past 3 months (χ2: 30.023, *p*: < 0.001)					
No	17,056	68.9 [67.7,70.1]	37,405	42.7 [42.1,43.4]	26.2
Yes	277	73.0 [66.2,78.9]	1074	51.4 [48.3,54.4]	21.7
Working status (χ2: 72.612, *p*: < 0.001)					
Not Working	2352	70.4 [67.8,72.9]	5047	57.0 [41.5,44.5]	13.3
Working	227	61.6 [52.8,69.7]	548	34.1 [31.0,37.3]	27.6
Ownership of bank account (χ2: 159.167, *p*: < 0.001)					
No	732	65.3 [60.5,69.8]	1483	34.4 [32.3,36.5]	30.9
Yes	1847	71.4 [68.4,74.1]	4112	45.5 [43.8,47.2]	25.9
Ownership of mobile phone (χ2: 456.470, *p*: < 0.001)					
No	1315	62.6 [59.3,65.8]	3740	38.5 [36.9,40.1]	24.2
Yes	1264	78.5 [75.4,81.4]	1855	51.1 [48.7,53.5]	27.4
Problem regarding getting medical help for self: getting permission to go (χ2: 1944.765, p: < 0.001)					
No problem	12,231	72.8 [71.6,74.1]	24,200	47.4 [46.6,48.2]	25.5
Big problem	1679	56.2 [53.0,59.3]	5738	35.0 [33.9,36.2]	21.1
Not a big problem	3423	64.0 [61.8,66.1]	8542	38.5 [37.4,39.5]	25.5
Problem regarding getting medical help for self: getting money needed for treatment (χ2: 3165.688, p: < 0.001)					
No problem	10,987	75.4 [74.1,76.6]	19,736	49.9 [49.1,50.8]	25.5
Big problem	2134	54.6 [51.7,57.5]	7643	35.0 [34.0,36.1]	19.6
Not a big problem	4211	63.3 [61.3,65.3]	11,100	39.2 [38.3,40.2]	24.1
Problem regarding getting medical help for self: distance of health facility (χ2: 3155.671, p: < 0.001)					
No problem	10,571	74.4 [73.0,75.7]	16,222	50.7 [49.8,51.7]	23.6
Big problem	2052	58.5 [55.8,61.2]	9215	36.8 [35.8,37.8]	21.8
Not a big problem	4711	63.5 [61.5,65.5]	13,042	40.0 [39.1,40.8]	23.6
Problem regarding getting medical help for self: transportation (χ2: 3358.624, *p*: < 0.001)					
No problem	11,113	74.7 [73.3,76.0]	16,897	50.6 [49.7,51.5]	24.1
Big problem	1764	56.5 [53.5,59.5]	8851	37.4 [36.4,38.4]	19.2
Not a big problem	4455	62.5 [60.5,64.5]	12,731	39.0 [38.1,40.0]	23.4
Use of hygienic materials*	**17,333**	**68.2 [67.7,70.2]**	**38,479**	**42.7 [42.3,43.6]**	**25.5**

N = sample size, CI = confidence interval, all percentages are weighted, # = women who used hygienic materials exclusively, χ2 values are for the total sample, pp = percentage points, * = exclusive use of sanitary napkins, locally prepared napkins, tampons, and menstrual cups.

The rural–urban gap in the use of hygienic materials was substantially higher among the unmarried adolescent as compared to their married counterparts (28.6 pp vs. 16.0 pp). While the proportion of women reporting use was higher among Hindu as compared to Muslims in both rural and urban areas, the rural–urban gap in the use was more pronounced among Hindus, with a significant 28 pp difference (urban: 71%, rural: 43%). The use was considerably higher among Others as compared to other social groups in both rural and urban areas. However, within each social category, the rural–urban gap was more or less same (range: 24–27 pp).

The rural–urban gap in the use was higher among those women who had discussed menstrual hygiene with health workers as compared to those who did not. The gap was higher among working women as comapred to their counter parts (27.6 pp vs 13.6 pp). The rural–urban gap in use of hygienic materials is higest in central and north-eastern region of India (more than 25 pp). On the other hand, the gap was relatively smaller in the southern (11 pp) and western (19 pp) of India.

### Rural–urban gap in the use across the states and UTs

**Figure 2:**
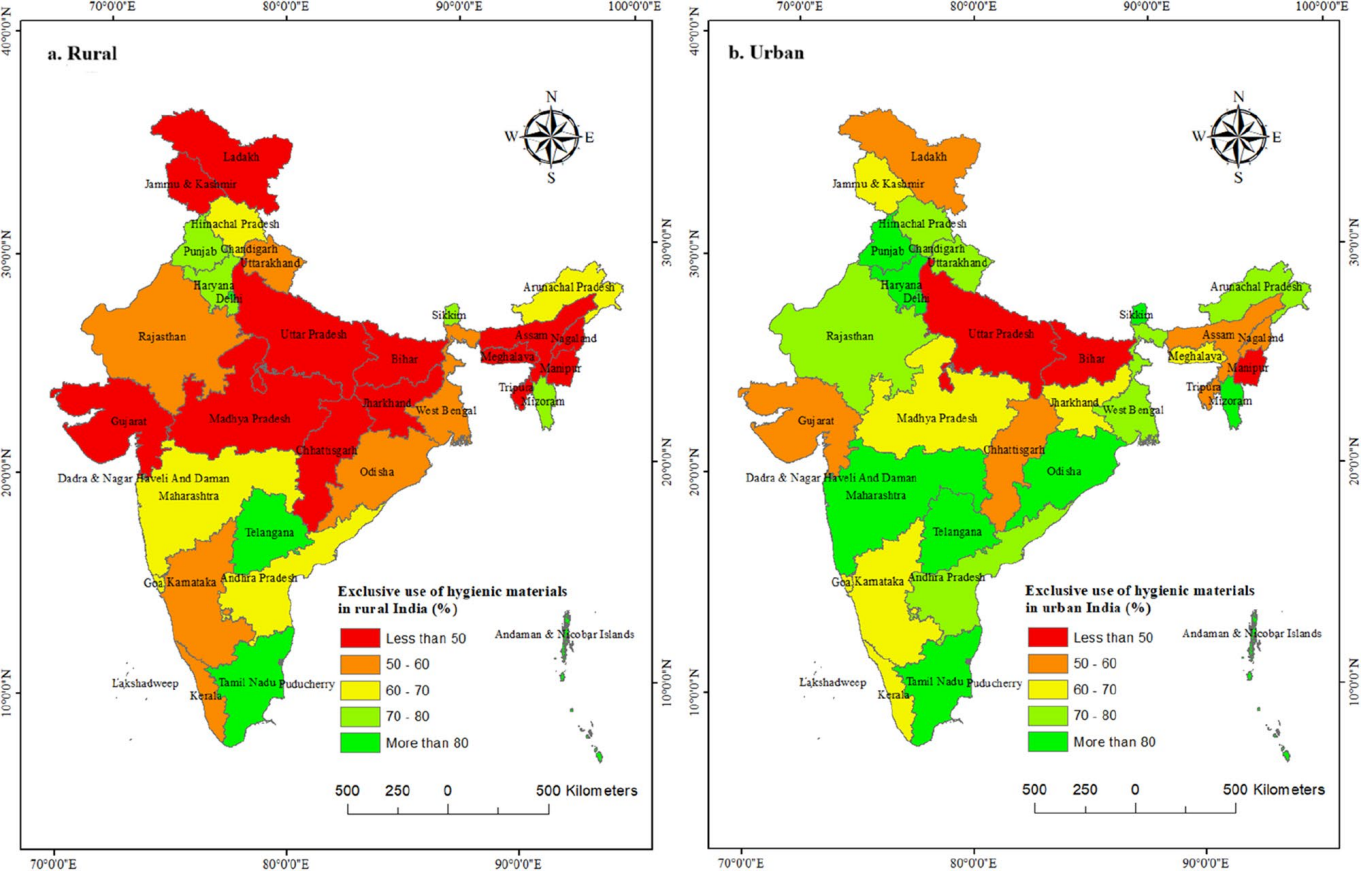
(**a**) Use of hygienic materials among adolescent women of rural India; (**b**) Use of hygienic materials among adolescent women of urban India, NFHS-5, 2019–21. Maps were created by authors using QGIS 3.32.3^44^ https://www.qgis.org/en/site/forusers/download.html#. Base maps (source or shape files) are authors own creation, and not taken/modified from any third party.

Figures 2a,b present the state- and UT-wise distribution of the use of hygienic materials among adolescent women in rural and urban India, respectively. On average, the proportion of adolescent women using hygienic materials is lower in rural than urban areas, across the different states. However, in three states (Bihar, Uttar Pradesh, and Manipur), almost half of the of adolescent women were not using hygienic materials in urban areas as well.

In both rural and urban India, among the bigger states, the highest use among adolescent women is identified in Tamil Nadu, followed by Telangana. On the other hand, in rural India, adolescent women of Uttar Pradesh, followed by Madhya Pradesh have reported lowest use of hygienic materials.

**Figure 3:**
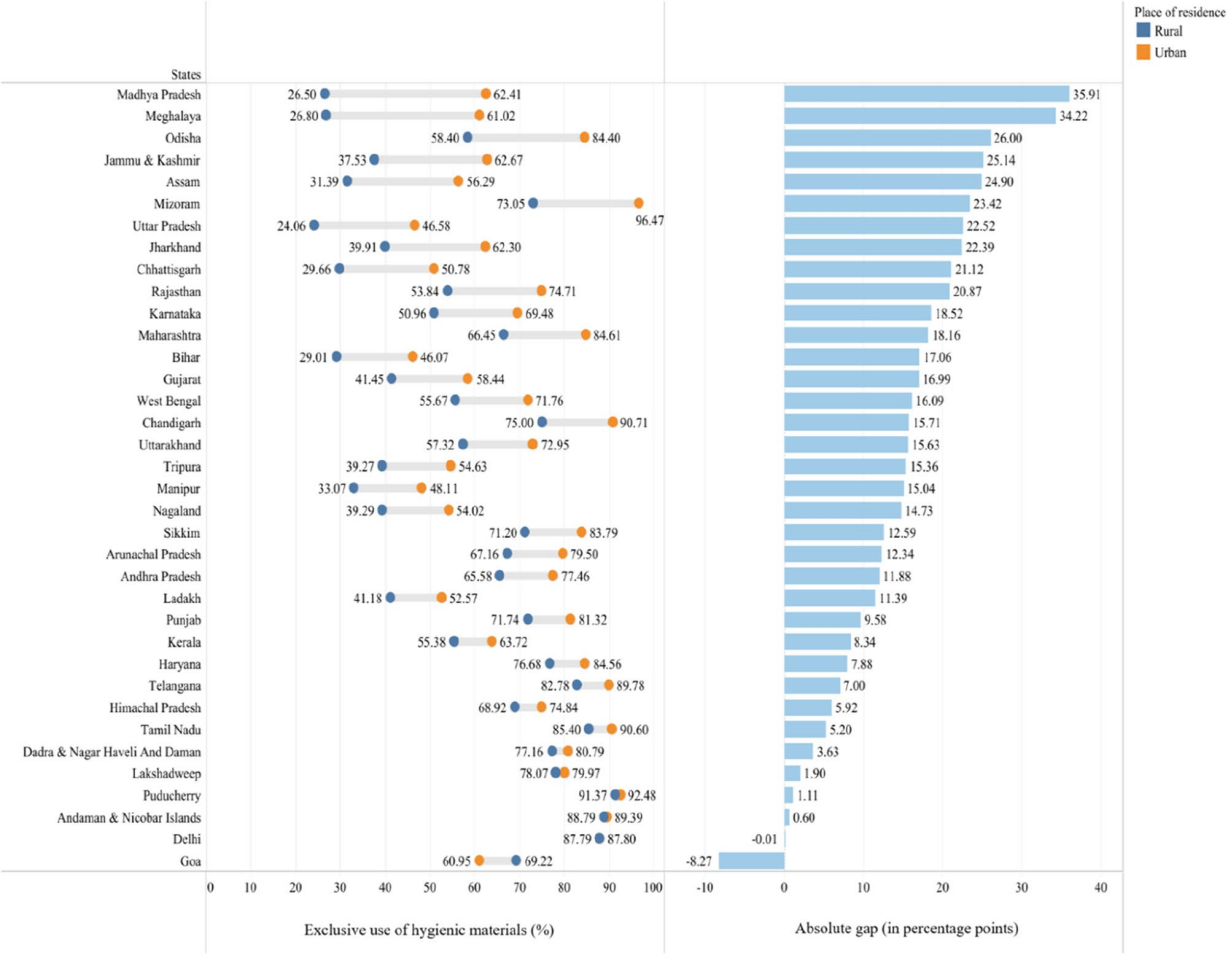
State-wise rural–urban gap in the use of hygienic materials among adolescent women of India, NFHS-5, 2019–21.

Figure 3 presents the rural–urban gap in use of hygienic materials among adolescent women across the states and UTs of India. In India, the average rural–urban gap in use of hygienic materials is 25 pp (rural: 43%; urban: 68%). The rural–urban gap in use of hygienic materials varied substantially across states and UTs with highest being Madhya Pradesh (30 pp) and lowest in Delhi where gap is (-0.01 pp).

In 10 out of the 28 states, the rural–urban gap in usage is notably high, exceeding 20 pp. These states include some of the empowered action group (EAG) states such as Madhya Pradesh, Odisha, Uttar Pradesh, Jharkhand, Chhattisgarh, and Rajasthan, as well as some northeastern states like Meghalaya, Assam, and Mizoram.

Conversely, there are states and UTs where the rural–urban gap in use is less than 10 pp. These areas primarily encompass states in northwestern India like Punjab, Haryana, Himachal Pradesh, and Delhi, as well as some southern states such as Kerala, Telangana, and Tamil Nadu. Additionally, there are four UTs (Dadra & Nagar Haveli and Daman, Lakshadweep, Puducherry, and Andaman & Nicobar Islands) where the difference in hygienic material usage between urban and rural women is negligible (less than 4 pp).

### Results of decomposition analysis

We use the Fairlie decomposition to break down the rural–urban gap in the use of hygienic materials among adolescent women and quantify the contribution of different factors explaining this gap.

The summary results of the decomposition analysis are presented in Table 3. Results indicate the use of hygienic materials for menstrual bloodstain is low among rural women than urban women. For instance, the probability of the use of hygienic materials among rural women is 0.42 compared to 0.68 for their urban counterparts. The result further indicates that almost 70% of such differences are explained by the factors included in the decomposition analysis.

**Table 3:** Summary result of Fairlie decomposition analysis showing the mean difference in the use of hygienic materials between urban and rural India, 2019–2021.

Total number of observation	1,14,805
Total number of observation (urban)	25,135
Total number of observation (rural)	89,670
Mean prediction for urban	0.6820
Mean prediction for rural	0.4273
Mean difference (urban–rural)	0.2547
Total explained	0.1769
Percentage explained	69.5
Percentage unexplained	30.5

Table 4 presents the decomposition analysis of the rural–urban gap in the use of hygienic materials for menstrual bloodstain prevention. To enhance interpretability, we have expressed the coefficients as percentages in our results. Over 90% of the explained gap is attributed to the differences in the distribution of only some selected predictors such as household wealth, region of residence, problem of getting money to get medical help for self, mass media exposure, and level of education.

**Table 4:** Contribution of factors explaining the urban–rural gap for the use of hygienic materials among adolescent women in India, 2019–2021.

Variables	Coefficient [95% CI]	% Contribution	*p*-value
Household wealth index	0.12289 [0.11264,0.13314]	69.4	< 0.001
Region of residence	0.02079 [0.01827,0.02332]	11.7	< 0.001
Problem regarding getting medical help for self: getting money needed for treatment	0.00990 [0.00718,0.01263]	5.6	< 0.001
Exposure to mass media	0.00876 [0.00483,0.01268]	4.9	< 0.001
Level of education	0.00771 [0.00621,0.00920]	4.4	< 0.001
Problem regarding getting medical help for self: transportation	0.00517 [0.00014,0.01020]	2.9	0.044
Problem regarding getting medical help for self: distance of health facility	0.00480 [−0.00014,0.00975]	2.7	0.057
Marital status	0.00197 [0.00051,0.00343]	1.1	0.008
Social groups	0.00176 [−0.00038,0.00390]	1.0	0.108
Ownership of mobile phone	0.00167 [0.00094,0.00240]	0.9	< 0.001
Problem regarding getting medical help for self: getting permission to go	0.00067 [−0.00089,0.00223]	0.4	0.400
Working status	0.00012 [−0.00012,0.00035]	0.1	0.342
Ownership of bank account	0.00000 [−0.00012,0.00012]	0.0	0.998
Age at menarche (in years)	−0.00002 [−0.00033,0.00029]	0.0	0.910
Discussed menstrual hygiene with healthcare workers in past 3 months	−0.00015 [−0.00060,0.00030]	−0.1	0.506
Religion	−0.00889 [−0.01021,−0.00756]	−5.0	< 0.001
Total	0.17713	100.0	

CI = Confidence Interval.

Household wealth is the main significant contributor explaining nearly 69% of the total gap in the use hygienic materials for menstrual bloodstain prevention between rural and urban India. Besides, region of residence is another contributor that explains nearly 12% of the gap. Mass media exposure and women's education level are among other significant contributors to the rural–urban gap in the use of hygienic materials among adolescent women in India. Problems related to getting money for medical treatment and transportation contribute nearly 8% of the rural–urban gap. The contribution of religion, ownership of mobile phone, and marital status of women is negligible.

## Discussion

The main objective of this study was to investigate how the rural–urban gap in the use of hygienic materials among adolescent women in India varies across different socioeconomic groups and geographies. Our findings indicate a substantial rural–urban gap, with urban adolescent women having a higher rate of use of hygienic material compared to their rural counterparts. Furthermore, this gap exhibited significant variations across different Indian states and UTs, with central (particularly EAG states) and northeastern states displaying a more pronounced rural–urban gap in the use than southern and northwestern states of the country. Additionally, the rural–urban gap in use varied according to the background characteristics of the women. For instance, the gap was wider among Hindu women, those with lower levels of education, and women from ST groups compared to others groups.

In addition, this study aimed to quantify the contribution of factors explaining the average gap in the use of hygienic materials. The results revealed that the majority of the gap is attributed to rural–urban differences in the household wealth, women’s education, mass media exposure, and problem of getting money to seek medical help, which could also affect affordability of hygienic materials. To the best of our knowledge, this is the first study in India at national level to identify the factors that underpin and explain the rural–urban gap in the use of hygienic materials among adolescent women.

The rural–urban disparity in household wealth has contributed significantly in widening the rural–urban gap in the use of hygienic materials among adolescent women. It must be noted here that according to the NFHS-5 data, close to half of rural adolescent women fall within the poor category (poorest and poorer quintiles). In contrast, urban areas report a markedly lower proportion, with only 13% of adolescent women classified as poor (as shown in Supplementary Table 1). This rural–urban disparity in poverty is consistent with findings from the 66th, 71st, and 75th rounds of the National Sample Survey, which underscore an unequal distribution of wealth concerning consumption and expenditure between rural and urban populations^48^. The association between household wealth and the use of hygienic materials is well-documented in existing literature^3,5,15,38^. As wealth of a household increases, so does the capacity and affordability to procure hygienic menstrual products or materials^3,27,49^. Also our study indicated that, in addition to factors like accessibility and availability, affordability has a significant association with the adoption of hygienic materials for menstrual hygiene. The findings of this study suggest that future initiatives aimed at reducing the rural–urban gap in the use of hygienic materials should prioritize the needs of the rural poor. This approach will enable them to narrow the substantial rural–urban gap in the use of hygineic materials.

Adolescent women’s education and mass media exposure also contributed significantly to the rural–urban gap in the use of hygienic materials. Notably, urban adolescents tend to have better access to education and attain higher levels of education compared to their rural counterparts, as indicated in Supplementary Table 1. Previous studies have consistently shown that more educated women are well-versed in hygienic material use and know the risk of unhygienic menstrual traditions^2,3,11,12,24,50^. The promotion of awareness regarding menstrual hygiene and health presents a formidable challenge, particularly in rural areas, where deeply rooted social and cultural taboos prevail^1^. Moreover, several previous studies have underscored the positive association of mass media exposure on the use of hygienic menstrual practices^1–3,24,51^. As mass media is a primary source of information on menstrual hygiene, women exposed to it frequently may use more hygienic materials. Thus, the use of mass media can be beneficial in spreading understanding and knowledge about menstrual hygiene^24,52^. Our result indicates that hygienic menstrual practices in urban areas are also high due to more mass media exposure in urban areas. Efforts should be made to create awareness through campaigns, and outreach programmes in rural areas, so that the rural women may also become aware of menstrual hygiene practices and the consequences of using unhygienic materials, e.g., increased likelihood of RTIs^2,39^.

Several programs have been instituted to address menstrual health and hygiene issues and promote the use of hygienic materials. For instance, since 2011, the MoHFW has been implementing a Menstrual Hygiene Scheme (MHS) across 107 rural districts, with support from the National Health Mission, aiming to raise awareness about menstrual hygiene, provide quality sanitary napkins, and ensure their proper disposal for adolescent girls aged 10–19^53,54^. Under MHS sanitary napkins are distributed by Accredited Social Health Activists (ASHA) at a reduced cost of INR 6 per pack^55^. However, this scheme currently covers only a fraction of India's adolescent girls, leaving a substantial portion underserved^56^. Additionally, the Swachh Bharat Abhiyan, led by the Ministry of Drinking Water and Sanitation, has formulated National Guidelines on Menstrual Hygiene Management (MHM) to promote better sanitation and hygiene practices in rural areas, but often faces challenges related to irregular and insufficient funding^55,57^. To achieve sustainability, it is vital to engage various stakeholders and ensure a consistent supply of subsidized sanitary products^2,58,59^. Some of these efforts are already underway. Since 2018, the Indian government introduced "Suvidha," 100% oxy-biodegradable sanitary napkins at a minimum price of Rs.1/-per pad, which are getting sold at *Pradhan Mantri Bhartiya Jan Aushadhi Kendras* (JAK), the government-owned subsidized rate pharmacy registered under the Pradhan Mantri Bhartiya Janaushadhi Pariyojana (PMBJP) campaign^2^. As JAK are mostly based in cities, it is imperative to actively promote and extend such initiatives to rural areas. This will contribute to making hygienic materials more affordable in rural regions. Unfortunately, the promotion and implementation of these government-supported programs are currently less prevalent in rural areas. Consequently, there is a pressing need for increased funding and resources to promote menstrual hygiene among rural adolescent women, considering the larger rural population.

Additionally, various state-specific policies like *Khushi* (Odisha), *Shuchi* (Karnataka), *Kishori Shakti Yojana* (Bihar and Uttar Pradesh) etc. exclusively focus on the menstrual health of rural adolescents, where governments distribute free or subsidized sanitary napkins to these young women^60–64^. Along with this several non-governmental organizations (NGOs) also support women by providing free or subsidized pads to rural adolescents at local level^65–69^. Despite concerted efforts at both the central and state levels, the rural–urban gap in use persists. Therefore, it is essential to conduct further research to understand the reasons behind the success and failures of these schemes. Furthermore, our findings underscore the need to focus on the rural poor, those with lower educational levels, and limited exposure to mass media, in order to effectively bridge this gap.

This study have certain limitations that warrant acknowledgment. To begin with, the NFHS-5 lacks data on various potential factors influencing hygienic material use, including myths, traditional beliefs, affordability, and the availability of such materials. Future research could consider incorporating these variables into the analysis to provide a more comprehensive understanding of menstrual hygiene practices. Additionally, it's important to recognize that menstrual hygiene remains a sensitive and often taboo subject among Indian women. This could introduce the possibility of social desirability bias during data collection, where respondents may provide answers they perceive as socially acceptable rather than reflecting their true practices and beliefs. Furthermore, given that the NFHS-5 dataset is cross-sectional, it is essential to acknowledge that causality between menstrual hygiene practices and their predictors cannot be definitively established. Further longitudinal studies would be valuable in exploring the causal relationships between these factors. Also, it's worth noting that cloth materials, if appropriately washed, dried, and stored, can also be considered hygienic for menstrual purposes. However, the dataset employed in this study does not include information pertaining to these specific practices. Subsequent research could delve into these aspects to gain a more comprehensive understanding of menstrual hygiene practices in different contexts.

## Conclusion

There is a significant rural–urban disparity in the use of hygienic materials among adolescent women in India. The study indicates that household wealth, women's education, and exposure to mass media largely contribute to this gap. To bridge this rural–urban divide in the use of hygienic materials among adolescent women in the country, it is recommended to raise awareness about menstrual hygiene through mass-media campaigns, especially among rural women. Additionally, educating women about the benefits of using hygienic materials during menstruation and providing subsidized or free hygienic products, especially to less educated and poor rural women, could reduce this gap in the near future.

### Supplementary Information

Supplementary Information.

## Data Availability

The study utilizes secondary sources of data that are freely available in the public domain through https://dhsprogram.com/methodology/survey/survey-display-541.cfm. Those who wish to access the data may register at the above link and thereafter can download the required data free of cost.

## References

[1] Garg R, Goyal S, Gupta S (2012). India moves towards menstrual hygiene: Subsidized sanitary napkins for rural adolescent girls - Issues and challenges. Matern. Child Health J..

[2] Ram U, Pradhan MR, Patel S, Ram F (2020). Factors associated with disposable menstrual absorbent use among young women in India. Int. Perspect. Sex. Reprod. Health.

[3] Roy A (2021). Prevalence and correlates of menstrual hygiene practices among young currently married women aged 15–24 years: an analysis from a nationally representative survey of India. Eur. J. Contracept. Reprod. Heal. Care.

[4] Roeckel, S., Cabrera-Clerget, A. & Yamakoshi, B. Guide to menstrual hygiene materials. *UNICEF* 6–36 (2019).

[5] Ram, U., Pradhan, M. R., Patel, S. & Ram, F. Factors associated with disposable menstrual absorbent use among young women in India. *Int. Perspect. Sex.* (2020) 10.1363/46e0320. 10.1363/46e0320 33108760

[6] Singh A, Chakrabarty M, Chowdhury S, Singh S (2022). Exclusive use of hygienic menstrual absorbents among rural adolescent women in India: A geospatial analysis. Clin. Epidemiol. Glob. Heal..

[7] Anton, B., Kim, W., Nair, A. & Wang, E. *Menstrual Hygiene Management- Evidence from the 6th Round of MICS*. *MICS Methodological Papers, No. 11* (2021).

[8] Das P (2021). Identifying risk factors for lower reproductive tract infections among women using reusable absorbents in Odisha, India. Int. J. Environ. Res. Public Health.

[9] Das P (2015). Menstrual hygiene practices, WASH access and the risk of urogenital infection in women from Odisha. India. PLoS One.

[10] Mahajan T (2019). Imperfect Information in Menstrual Health and the Role of Informed Choice. Indian J. Gend. Stud..

[11] Anand E, Singh J, Unisa S (2015). Menstrual hygiene practices and its association with reproductive tract infections and abnormal vaginal discharge among women in India. Sex. Reprod. Healthc..

[12] Vishwakarma D, Puri P, Sharma SK (2020). Interlinking menstrual hygiene with women’s empowerment and reproductive tract infections: evidence from India. Clin. Epidemiol. Glob. Heal..

[13] Torondel B (2018). Association between unhygienic menstrual management practices and prevalence. BMC Infect. Dis..

[14] USAID. *Global strategy to empower adolescent girls*. https://www.state.gov/u-s-global-strategy-to-empower-adolescent-girls/ (2016).

[15] Singh A (2022). Menstrual hygiene practices among adolescent women in rural India: a cross-sectional study. BMC Public Health.

[16] Rani PS (2014). Knowledge and practices of menstrual hygiene among married adolescents and young women in Chittoor District of Andra Pradesh: India. IOSR J. Nurs. Heal. Sci..

[17] Thakre SB (2011). Menstrual hygiene: Knowledge and practice among adolescent school girls of Saoner. Nagpur District. J. Clin. Diagnostic Res..

[18] Malhotra A, Coli S, Coates S, Mosquera-Vasquez M (2016). Factors associated with knowledge, attitudes, and hygiene practices during menstruation among adolescent girls in Uttar Pradesh. Waterlines.

[19] Goli, S., Sharif, N., Paul, S. & Salve, P. S. Geographical disparity and socio-demographic correlates of menstrual absorbent use in India: A cross-sectional study of girls aged 15–24 years. *Child. Youth Serv. Rev.* (2020).

[20] Sumpter, C. & Torondel, B. A Systematic Review of the Health and Social Effects of Menstrual Hygiene Management. *PLoS One* **8**, (2013). 10.1371/journal.pone.0062004 PMC3637379 23637945

[21] Ramaiya, A. ‘Time of the month’: A mixed-method study to understand and improve Menstrual Health and Hygiene Management in Rural North India. *ProQuest Diss. Theses* 239 (2018).

[22] Sommer, M. *et al.* How addressing menstrual health and hygiene may enable progress across the Sustainable Development Goals. *Glob. Heal.* **14**, 1920315 (2021). 10.1080/16549716.2021.1920315 PMC8253211 34190034

[23] Ghosh, P. Determinants of Menstrual Hygiene Management among Young Indian Women : An investigation based on the National Family Health Survey 2015–16 Determinants of Menstrual Hygiene Management among Young Indian Women : An investigation based on the National Fami. (2021).

[24] Chauhan S (2021). Examining the predictors of use of sanitary napkins among adolescent girls: A multi-level approach. PLoS One.

[25] Singh A, Chakrabarty M (2023). Spatial heterogeneity in the exclusive use of hygienic materials during menstruation among women in urban India. PeerJ.

[26] Singh A, Chakrabarty M, Chandra R (2023). Intra-urban differentials in the exclusive use of hygienic methods during menstruation among young women in India. PLOS Glob. Public Heal..

[27] Babbar, K. & Garikipati, S. What socio-demographic factors support disposable vs. sustainable menstrual choices? Evidence from India’s National Family Health Survey-5. *PLoS One* **18**, e0290350 (2023). 10.1371/journal.pone.0290350 PMC10434932 37590271

[28] Babbar, K. Development and validation of the menstrual health and hygiene ( MHH ) scale for adolescent girls and teachers : associating MHH needs , practices , beliefs and experiences of adolescent girls and teachers with student engagement. (IIM Ahmedabad, 2022).

[29] Martin, J., Babbar, K. & Maschette, U. Menstrual health for all requires wider high level commitment. *BMJ* 1–2 (2022) 10.1136/bmj.o2222. 10.1136/bmj.o2222 36126991

[30] Anand E, Unisa S, Singh J (2015). Menstrual hygiene management among young unmarried women in India. Soc. Sci. Spectr..

[31] Almeida-Velasco A, Sivakami M (2019). Menstrual hygiene management and reproductive tract infections: A comparison between rural and urban India. Waterlines.

[32] Valvaikar K, Shah H (2016). An urban-rural comparison of menstrual pattern and menstrual problems among school-going girls. Int. J. Med. Sci. Public Heal..

[33] Maharana B (2022). What explains the rural-urban gap in the use of hygienic methods of menstrual protection among youth in the east Indian state of Bihar?. Indian J. Commun. Med..

[34] Paria, B., Bhattacharyya, A. & Das, S. A comparative study on menstrual hygiene among urban and rural adolescent girls of West Bengal. *J. Fam. Med.* (2014). 10.4103/2249-4863.148131 PMC4311354 25657955

[35] Rossouw L, Ross H (2021). Understanding period poverty: Socio-economic inequalities in menstrual hygiene management in eight low-and middle-income countries. Int. J. Environ. Res. Public Health.

[36] Chauhan S, Kumar P, Marbaniang SP, Srivastava S, Patel R (2022). Prevalence and predictors of anaemia among adolescents in Bihar and Uttar Pradesh. India. Sci. Rep..

[37] International Institute for Population Sciences (IIPS) & ICF. *National Family Health Survey (NFHS-5), 2019–21 India Report*. (2021).

[38] Singh A (2022). Wealth-based inequality in the exclusive use of hygienic materials during menstruation among young women in urban India. PLoS One.

[39] Dasgupta A, Sarkar M (2008). Menstrual hygiene: How hygienic is the adolescent girl?. Indian J. Commun. Med..

[40] Kathuria B, Raj S (2018). Effects of socio-economic conditions on usage of hygienic method of menstrual protection among young women in EAG states of India. AJHM ADMAA Amity J. Healthc. Manag..

[41] Filmer, D. & Pritchett, L. Estimating Wealth Effects without Expenditure Data-or Tears : An Application to Educational Enrollments in States of India Author ( s ): Deon Filmer and Lant H. Pritchett Published by: Springer on behalf of the Population Association of America Stable U. *Demography* **38**, 115–132 (2001). 10.1353/dem.2001.0003 11227840

[42] International Institute for Population Sciences (2020). National Family Health Survey - 5 2019–21. Minist. Heal. Fam. Welf. Natl..

[43] StataCorp.  (2019). Stata Statistical Software: Release 16.

[44] QGIS.org. QGIS Geographic Information System. *Open Source Geospatial Foundation Project* https://www.qgis.org/en/site/forusers/download.html# (2023).

[45] Fairlie RW (2006). An extension of the Blinder-Oaxaca decomposition technique to logit and probit models. J. Econ. Soc. Meas..

[46] Fairlie R (2005). An extension of the blinder-oaxaca decomposition technique to logit and probit models. J. Econ. Soc. Meas..

[47] Kim JH (2019). Multicollinearity and misleading statistical results. Korean J. Anesthesiol..

[48] Download Reports | Ministry of Statistics and Program Implementation | Government Of India.

[49] Chakrabarty M, Singh A, Singh S, Tripathi P (2023). Spatiotemporal change in socioeconomic inequality in hygienic menstrual product use among adolescent girls in India during 2015–2019. Int. J. Equity Health.

[50] Singh A, Chakrabarty M, Chowdhury S, Singh S (2022). Exclusive use of hygienic menstrual absorbents among rural adolescent women in India: A geospatial analysis. Clin. Epidemiol. Glob. Heal..

[51] Babbar, K., Saluja, D. & Sivakami, M. How socio-demographic and mass media factors affect sanitary item usage among women in rural and urban India. *Waterlines* (2021).

[52] Malhotra A, Coli S, Coates S, Mosquera-Vasquez M (2016). Factors associated with knowledge, attitudes, and hygiene practices during menstruation among adolescent girls in Uttar Pradesh. Waterlines.

[53] MoHFW. Scheme for Promotion of Menstrual Hygiene among Girls in Rural India. (2016).

[54] Ministry of Health & Family Welfare. *National health mission: Menstrual hygiene scheme*. (2017).

[55] Ministry of Women and Child Development. *Scheme for Promotion of Menstrual Hygiene*. https://pib.gov.in/PressReleasePage.aspx?PRID=1846147 (2022).

[56] UNESCO. *Menstrual Health and Hygiene Management: Survey and Gap Analysis Report*. https://en.unesco.org/fieldoffice/newdelhi For (2023).

[57] Smith, A. D., Muli, A., Schwab, K. J. & Hennegan, J. National monitoring for menstrual health and hygiene: is the type of menstrual material used indicative of needs across 10 countries? *Int. J.* (2020). 10.3390/ijerph17082633 PMC7215803 32290529

[58] Goyal V (2016). Scope and opportunities for menstrual health and hygiene products in India. Int. Res. J. Soc. Sci. E-ISSN.

[59] Bhattacharya S, Singh A (2016). How effective is the Menstrual Hygiene Scheme? An evaluation study from North India. Int. J. Commun. Med. Public Heal..

[60] Buradikatti, K. With Shuchi Scheme yet to restart , adolescent girl students have a tough time in rural Karnataka. *The Hindu* (2022).

[61] Department of Health and Family Welfare. Khusi. *Odisha State Medical Corporation, Government of Odisha* https://khushi.nic.in/ (2022).

[62] Ministry of Women & Child Development. *Kishori Shakti Yojana*. https://wcd.nic.in/kishori-shakti-yojana (2017).

[63] PMY Team. Maharashtra Kishori Shakti Yojana 2023. *Women & Child Development Department* https://pmmodiyojana.in/maharashtra-kishori-shakti-yojana/ (2023).

[64] PM Jan Dhan Yojana. *Kishori Suraksha Yojana in Uttar Pradesh - PM Jan Dhan Yojana*. https://pmjandhanyojana.co.in/kishori-suraksha-uttar-pradesh-gov/ (2015).

[65] Dabriwal, S. & Rai Gupta, A. Project Baala. 1–9 (2021).

[66] Agarwal, R., Shah, R. & Vatsa, U. Sangini - Affordable Sanitary Pads for Rural Girls & Women. *Wheels Global Foundation* 1–6 https://wheelsglobal.org/sevak-3/ (2023).

[67] Donatekart. Empowering Women : Promoting Period Hygiene through Sanitary Pad Distribution in Tribal and Rural Areas of India by Donating 250 Rupees per year for one woman. 1–8 (2023).

[68] Helping Hand India NGO. Sanitary Pads Distribution For Poor Women.

[69] Kasondra, R. J. Sponsor sanitary napkins for rural women- Give India. *Gram Vikas Trust* 1–15 https://www.giveindia.org/program/sponsor-sanitary-napkins-for-rural-women (2023).

