# Supplementary material for: Decomposing the rural–urban gap in hygienic material use during menstruation among adolescent women in India
Source: Sci Rep. 2023 Dec 16;13:22427. doi: 10.1038/s41598-023-49682-1 (PMC10725416; doi:10.1038/s41598-023-49682-1)
Supplement: Supplementary file 1 — Supplementary Information. [file 41598_2023_49682_MOESM1_ESM.docx]

**Appendix 1: Fairlie decomposition**

This decomposition calculates the difference in the probability of an outcome between two groups and quantifies the contribution of group variations in the independent variables to the outcome differential (here, the two groups are rural/urban).

The equation for Rural-Urban decomposition for a nonlinear equation $\text{y=F}\left( x\beta\right)$ can be written as (Fairlie, 2005):

$y^{-U}-y^{-R}=\left[ \sum_{i=1}^{N^{U}} \frac{F\left( x_{i}^{U}\beta^{U} \right)}{N^{U}} \right.-\left. \sum_{i=1}^{N^{R}} \frac{F\left( x_{i}^{R}\beta^{U} \right)}{N^{R}} \right] +\left[ \sum_{i=1}^{N^{R}} \frac{F\left( x_{i}^{R}\beta^{U} \right)}{N^{R}} \right.-\left. \sum_{i=1}^{N^{R}} \frac{F\left( x_{i}^{R}\beta^{R} \right)}{N^{R}} \right]$

Here N^j^ is the sample size for any group j (here, j may be U or R, which represents urban and rural, respectively), Y^j^ is the average probability of the binary outcome of the interest group j and F is the cumulative distribution function from the logistic distribution. Finally, β is the coefficient estimate for group j. The first term in the brackets represents the portion of the group differences due to group differences in the distribution of the independent variable's characteristics, often known as the "explained part." The second term denotes the portion of y that is determined by differences in group processes. The second term additionally accounts for the fraction of the group gap caused by differences in immeasurable or unobserved endowments between groups.

To get the total contribution explained by each predictor, we must calculate two sets of predicted probabilities, one for urban and one for rural, and then subtract the average values of the two. However, obtaining the contribution of a specific predictor is not straightforward.

Because the sample sizes of the two groups differ, we must do a regression for pooled data (rural and urban population combined) and calculate the predicted probability for each rural and urban population observation in the sample. Because the rural population sample is larger than the urban population sample, a random subsample of the rural population of the same size as the urban sample should be drawn. Each observation in the rural and urban population samples is then ranked by predicted probabilities and matched by their respective rankings.

This approach pairs urban women with characteristics that place them at the bottom (top) of their distribution with rural women with characteristics that put them at the bottom (top) of their distribution.

Now assume that rural and urban samples are equal, i.e., N^U^= N^R,^ and a natural one-to-one matching of urban and rural population observations exists. Also, consider that there are two independent variables (x_1_ & x_2_) to explain the rural-urban gap in the use of hygienic materials among adolescent women.

Using coefficient estimates from a logit regression for a pooled sample$(\hat{\beta}^{*})$, the independent contribution of x_1_ to the group gap can then be expressed as:

$$\frac{1}{N^{R}}\sum_{i=1}^{N^{R}} F\left( \hat{\alpha}+ x_{1i}^{U}\hat{\beta}_{1}^{*}+x_{2i}^{U}\hat{\beta}_{2}^{*} \right)-F\left( \hat{\alpha}+ x_{1i}^{R}\hat{\beta}_{1}^{*}+x_{2i}^{U}\hat{\beta}_{2}^{*} \right)$$

Similarly, the gap due to x_2_ can be expressed as:

$$\frac{1}{N^{R}}\sum_{i=1}^{N^{R}} F\left( \hat{\alpha}+ x_{1i}^{R}\hat{\beta}_{1}^{*}+x_{2i}^{U}\hat{\beta}_{2}^{*} \right)-F\left( \hat{\alpha}+ x_{1i}^{R}\hat{\beta}_{1}^{*}+x_{2i}^{R}\hat{\beta}_{2}^{*} \right)$$

Thus, the contribution of each variable to the gap is therefore equal to the change in average predicted probability from replacing urban distribution with rural population distribution while holding the distributions of the other variables constant.

However, in practice, the assumption of equal sample size is rarely valid. Because the rural population sample is significantly larger, many random subsamples of rural women (equivalent in size to the whole urban sample) are generated to match each to the urban sample and calculate separate decomposition. Finally, the mean value of these individual decomposition estimations is used as an estimated decomposition for the remaining population sample. We performed 1000 replications of this decomposition and reported the average result. It should be mentioned that increasing the number of replications improves the consistency of the results.

**References**

Fairlie R. 2005. An Extension of the Blinder-Oaxaca Decomposition Technique to Logit and Probit Models. *Journal of Economic and Social Measurement* 30:305–316.

**Supplementary Table 1: Percentage distribution of adolescent women by background characteristics in urban and rural India, NFHS-5 (2019-21)**

| **Background characteristics** | **Urban** | | **Rural** | | **Total** | |
| --- | --- | --- | --- | --- | --- | --- |
|  | **Frequency (N=25,135)** | **%** | **Frequency (N=89,670)** | **%** | **Frequency (N=114,805)** | **%** |
| **Age at menarche (in years)** | |  |  |  |  |  |
| ≤12 | 5,409 | 21.52 | 17,031 | 18.99 | 22,440 | 19.55 |
| 13-15 | 19,117 | 76.06 | 70,131 | 78.21 | 89,248 | 77.74 |
| ≥16 | 609 | 2.42 | 2,508 | 2.80 | 3,117 | 2.72 |
| **Marital status** |  |  |  |  |  |  |
| Not married | 23,499 | 93.49 | 78,173 | 87.18 | 1,01,672 | 88.56 |
| Married before 18 years | 806 | 3.21 | 6,489 | 7.24 | 7,295 | 6.35 |
| Married on or after 18 years | 830 | 3.30 | 5,008 | 5.58 | 5,838 | 5.09 |
| **Level of education** |  |  |  |  |  |  |
| No education | 585 | 2.33 | 4,505 | 5.02 | 5,090 | 4.43 |
| Primary | 832 | 3.31 | 5,188 | 5.79 | 6,020 | 5.24 |
| Secondary | 21,227 | 84.45 | 74,992 | 83.63 | 96,219 | 83.81 |
| Higher | 2,491 | 9.91 | 4,985 | 5.56 | 7,476 | 6.51 |
| **Religion** |  |  |  |  |  |  |
| Hindu | 17,786 | 70.76 | 70,819 | 78.98 | 88,605 | 77.18 |
| Muslim | 4,673 | 18.59 | 9,099 | 10.15 | 13,772 | 12.00 |
| Christian | 1,726 | 6.87 | 5,880 | 6.56 | 7,606 | 6.63 |
| Others | 950 | 3.78 | 3,872 | 4.32 | 4,822 | 4.20 |
| **Social group** |  |  |  |  |  |  |
| SC | 5,087 | 20.24 | 19,813 | 22.10 | 24,900 | 21.69 |
| ST | 2,848 | 11.33 | 19,244 | 21.46 | 22,092 | 19.24 |
| OBC | 10,896 | 43.35 | 36,800 | 41.04 | 47,696 | 41.55 |
| Other | 6,304 | 25.08 | 13,813 | 15.40 | 20,117 | 17.52 |
| **Household wealth index** | |  |  |  |  |  |
| Poorest | 968 | 3.85 | 26,254 | 29.28 | 27,222 | 23.71 |
| Poorer | 2,277 | 9.06 | 25,874 | 28.85 | 28,151 | 24.52 |
| Middle | 4,700 | 18.70 | 19,611 | 21.87 | 24,311 | 21.18 |
| Richer | 7,649 | 30.43 | 12,264 | 13.68 | 19,913 | 17.35 |
| Richest | 9,541 | 37.96 | 5,667 | 6.32 | 15,208 | 13.25 |
| **Region of residence** |  |  |  |  |  |  |
| North | 5,913 | 23.52 | 16,921 | 18.87 | 22,834 | 19.89 |
| Central | 5,656 | 22.50 | 27,166 | 30.30 | 32,822 | 28.59 |
| East | 3,054 | 12.15 | 18,213 | 20.31 | 21,267 | 18.52 |
| West | 3,016 | 12.00 | 7,162 | 7.99 | 10,178 | 8.87 |
| Southern | 4,975 | 19.79 | 9,570 | 10.67 | 14,545 | 12.67 |
| North-east | 2,521 | 10.03 | 10,638 | 11.86 | 13,159 | 11.46 |
| **Exposure to mass media** | |  |  |  |  |  |
| No exposure | 1,959 | 7.79 | 20,670 | 23.05 | 22,629 | 19.71 |
| Low exposure | 10,917 | 43.43 | 43,092 | 48.06 | 54,009 | 47.04 |
| Medium exposure | 10,558 | 42.01 | 22,582 | 25.18 | 33,140 | 28.87 |
| High exposure | 1,701 | 6.77 | 3,326 | 3.71 | 5,027 | 4.38 |
| **Discussed menstrual hygiene with healthcare workers in past 3 months** | | | | |  |  |
| No | 24,785 | 98.61 | 87,605 | 97.70 | 1,12,390 | 97.90 |
| Yes | 350 | 1.39 | 2,065 | 2.30 | 2,415 | 2.10 |
| **Working status** |  |  |  |  |  |  |
| Not Working | 3,430 | 13.65 | 11,631 | 12.97 | 15,061 | 13.12 |
| Working | 326 | 1.30 | 1,733 | 1.93 | 2,059 | 1.79 |
| Question not asked | 21,379 | 85.06 | 76,306 | 85.10 | 97,685 | 85.09 |
| **Ownership of bank account** | |  |  |  |  |  |
| No | 1,137 | 4.52 | 4,519 | 5.04 | 5,656 | 4.93 |
| Yes | 2,619 | 10.42 | 8,845 | 9.86 | 11,464 | 9.99 |
| Question not asked | 21,379 | 85.06 | 76,306 | 85.10 | 97,685 | 85.09 |
| **Ownership of mobile phone** | | | |  |  |  |
| No | 2,048 | 8.15 | 9,277 | 10.35 | 11,325 | 9.86 |
| Yes | 1,708 | 6.80 | 4,087 | 4.56 | 5,795 | 5.05 |
| Question not asked | 21,379 | 85.06 | 76,306 | 85.10 | 97,685 | 85.09 |
| **Problem regarding getting medical help for self: getting permission to go** | | | | | |  |
| No problem | 16,676 | 66.35 | 51,579 | 57.52 | 68,255 | 59.45 |
| Big problem | 3,059 | 12.17 | 16,021 | 17.87 | 19,080 | 16.62 |
| Not a big problem | 5,400 | 21.48 | 22,070 | 24.61 | 27,470 | 23.93 |
| **Problem regarding getting medical help for self: getting money needed for treatment** | | | | |  |  |
| No problem | 14,281 | 56.82 | 38,126 | 42.52 | 52,407 | 45.65 |
| Big problem | 3,973 | 15.81 | 22,564 | 25.16 | 26,537 | 23.11 |
| Not a big problem | 6,881 | 27.38 | 28,980 | 32.32 | 35,861 | 31.24 |
| **Problem regarding getting medical help for self: distance of health facility** | | | | |  |  |
| No problem | 13,869 | 55.18 | 30,141 | 33.61 | 44,010 | 38.33 |
| Big problem | 3,729 | 14.84 | 27,083 | 30.20 | 30,812 | 26.84 |
| Not a big problem | 7,537 | 29.99 | 32,446 | 36.18 | 39,983 | 34.83 |
| **Problem regarding getting medical help for self: transportation** | | | | |  |  |
| No problem | 14,428 | 57.40 | 31,485 | 35.11 | 45,913 | 39.99 |
| Big problem | 3,353 | 13.34 | 25,785 | 28.76 | 29,138 | 25.38 |
| Not a big problem | 7,354 | 29.26 | 32,400 | 36.13 | 39,754 | 34.63 |

Note: N= sample size, all percentages are weighted

**Supplementary Table 2: Binary logistic regression (coefficient and p-values) showing the factors associated with use of hygienic materials among adolescent women in urban and rural India and for pooled data (rural and urban both), NFHS-5 (2019-21)**

| **Background characteristics** | **Urban** | | **Rural** | | **India (pooled)** | |
| --- | --- | --- | --- | --- | --- | --- |
|  | **Odds Ratio** | **p-value** | **Odds Ratio** | **p-value** | **Odds Ratio** | **p-value** |
| **Age at menarche (in years)** | | | | | | |
| ≤12 |  |  |  |  |  |  |
| 13-15 | 1.00 [0.93,1.07] | 0.967 | 1.04 [1.00,1.08] | 0.042 | 1.02 [0.99,1.06] | 0.181 |
| ≥16 | 1.22 [1.00,1.49] | 0.055 | 1.45 [1.32,1.59] | <0.001 | 1.38 [1.27,1.50] | <0.001 |
| **Marital status** | | | | | | |
| Not married® |  |  |  |  |  |  |
| Married before 18 years | 0.83 [0.71,0.97] | 0.019 | 0.93 [0.88,0.99] | 0.022 | 0.91 [0.86,0.96] | <0.001 |
| Married on or after 18 years | 0.89 [0.76,1.04] | 0.153 | 1.09 [1.02,1.16] | 0.012 | 1.03 [0.97,1.09] | 0.326 |
| **Level of education** | | | | | | |
| No education® |  |  |  |  |  |  |
| Primary | 1.15 [0.91,1.46] | 0.244 | 1.28 [1.14,1.43] | <0.001 | 1.26 [1.14,1.39] | <0.001 |
| Secondary | 2.05 [1.70,2.49] | <0.001 | 2.57 [2.35,2.80] | <0.001 | 2.42 [2.24,2.62] | <0.001 |
| Higher | 2.27 [1.83,2.83] | <0.001 | 3.45 [3.10,3.84] | <0.001 | 3.05 [2.77,3.35] | <0.001 |
| **Religion** | | | | | | |
| Hindu® |  |  |  |  |  |  |
| Muslim | 0.56 [0.52,0.60] | <0.001 | 0.62 [0.58,0.65] | <0.001 | 0.62 [0.59,0.65] | <0.001 |
| Christian | 1.12 [0.95,1.31] | 0.179 | 1.01 [0.94,1.09] | 0.805 | 1.04 [0.97,1.11] | 0.292 |
| Others | 1.49 [1.24,1.79] | <0.001 | 1.44 [1.34,1.55] | <0.001 | 1.39 [1.30,1.49] | <0.001 |
| **Social group** | | | | | | |
| SC® |  |  |  |  |  |  |
| ST | 1.10 [0.97,1.25] | 0.126 | 1.02 [0.97,1.07] | 0.534 | 1.01 [0.97,1.06] | 0.538 |
| OBC | 0.88 [0.81,0.95] | 0.001 | 0.94 [0.90,0.98] | 0.002 | 0.91 [0.88,0.94] | <0.001 |
| Other | 1.24 [1.13,1.36] | <0.001 | 1.08 [1.02,1.13] | 0.005 | 1.10 [1.05,1.15] | <0.001 |
| **Household wealth index** |  |  |  |  |  |  |
| Poorest® |  |  |  |  |  |  |
| Poorer | 1.48 [1.26,1.75] | <0.001 | 1.44 [1.38,1.50] | <0.001 | 1.47 [1.42,1.53] | <0.001 |
| Middle | 2.03 [1.74,2.38] | <0.001 | 2.02 [1.93,2.12] | <0.001 | 2.16 [2.07,2.25] | <0.001 |
| Richer | 3.09 [2.65,3.61] | <0.001 | 2.72 [2.58,2.87] | <0.001 | 3.18 [3.04,3.34] | <0.001 |
| Richest | 5.12 [4.36,6.01] | <0.001 | 4.11 [3.81,4.42] | <0.001 | 5.62 [5.31,5.94] | <0.001 |
| **Region of residence** | | | | | | |
| North® |  |  |  |  |  |  |
| Central | 0.40 [0.36,0.43] | <0.001 | 0.34 [0.33,0.36] | <0.001 | 0.36 [0.35,0.38] | <0.001 |
| East | 0.84 [0.75,0.94] | 0.002 | 0.85 [0.81,0.89] | <0.001 | 0.88 [0.84,0.92] | <0.001 |
| West | 0.65 [0.59,0.72] | <0.001 | 0.93 [0.88,0.99] | 0.017 | 0.89 [0.84,0.93] | <0.001 |
| Southern | 1.26 [1.14,1.40] | <0.001 | 1.74 [1.64,1.84] | <0.001 | 1.65 [1.57,1.73] | <0.001 |
| North-east | 0.75 [0.65,0.87] | <0.001 | 0.79 [0.74,0.84] | <0.001 | 0.82 [0.77,0.86] | <0.001 |
| **Exposure to mass media** | |  |  |  |  |  |
| No exposure® |  |  |  |  |  |  |
| Low exposure | 1.27 [1.14,1.41] | <0.001 | 1.24 [1.19,1.29] | <0.001 | 1.25 [1.20,1.30] | <0.001 |
| Medium exposure | 1.30 [1.16,1.46] | <0.001 | 1.34 [1.28,1.40] | <0.001 | 1.35 [1.29,1.41] | <0.001 |
| High exposure | 1.27 [1.09,1.49] | 0.002 | 1.22 [1.12,1.32] | <0.001 | 1.25 [1.17,1.35] | <0.001 |
| **Discussed menstrual hygiene with healthcare workers in past 3 months** | | | | | | |
| No® |  |  |  |  |  |  |
| Yes | 1.09 [0.85,1.40] | 0.510 | 1.04 [0.95,1.14] | 0.414 | 1.02 [0.94,1.12] | 0.607 |
| **Working status** | | | | | | |
| Not working® |  |  |  |  |  |  |
| Working | 0.79 [0.62,1.03] | 0.077 | 0.75 [0.67,0.85] | <0.001 | 0.77 [0.70,0.86] | <0.001 |
| **Ownership of bank account** | | | | | | |
| No® |  |  |  |  |  |  |
| Yes | 1.00 [0.85,1.18] | 0.998 | 1.1 [1.02,1.20] | 0.018 | 1.07 [1.00,1.15] | 0.061 |
| **Ownership of mobile phone** | | | | | | |
| No® |  |  |  |  |  |  |
| Yes | 1.44 [1.23,1.69] | <0.001 | 1.21 [1.11,1.31] | <0.001 | 1.28 [1.19,1.37] | <0.001 |
| **Problem regarding getting medical help for self: getting permission to go** | | | | | | |
| No problem® |  |  |  |  |  |  |
| Big problem | 0.96 [0.86,1.07] | 0.445 | 1.00 [0.95,1.05] | 0.981 | 0.99 [0.95,1.04] | 0.701 |
| Not a big problem | 0.97 [0.90,1.06] | 0.524 | 0.96 [0.92,1.00] | 0.035 | 0.96 [0.93,1.00] | 0.030 |
| **Problem regarding getting medical help for self: getting money needed for treatment** | | | | | | |
| No problem® |  |  |  |  |  |  |
| Big problem | 0.70 [0.63,0.78] | <0.001 | 0.79 [0.75,0.83] | <0.001 | 0.79 [0.76,0.83] | <0.001 |
| Not a big problem | 0.78 [0.72,0.85] | <0.001 | 0.83 [0.79,0.86] | <0.001 | 0.83 [0.80,0.86] | <0.001 |
| **Problem regarding getting medical help for self: distance of health facility** | | | | | | |
| No problem**®** |  |  |  |  |  |  |
| Big problem | 0.89 [0.79,1.02] | 0.084 | 0.88 [0.83,0.93] | <0.001 | 0.85 [0.81,0.90] | <0.001 |
| Not a big problem | 0.92 [0.84,1.01] | 0.068 | 0.94 [0.90,0.99] | 0.015 | 0.92 [0.88,0.96] | <0.001 |
| **Problem regarding getting medical help for self: transportation** | | | | | | |
| No problem® |  |  |  |  |  |  |
| Big problem | 0.89 [0.78,1.01] | 0.076 | 0.91 [0.86,0.97] | 0.002 | 0.90 [0.85,0.94] | <0.001 |
| Not a big problem | 0.91 [0.84,1.00] | 0.045 | 0.87 [0.83,0.91] | <0.001 | 0.87 [0.84,0.91] | <0.001 |

Note: **®=** reference category

**Supplementary Table 3: Variance Inflation Factors (VIF)**

| **Variables** | **VIF** | **1/VIF** |
| --- | --- | --- |
| **Marital status** |  |  |
| Not married® |  |  |
| Married <18 years | 1.06 | 0.95 |
| Married =>18 years | 1.02 | 0.98 |
| **Level of education** |  |  |
| No education® |  |  |
| Primary | 2.09 | 0.48 |
| Secondary | 3.56 | 0.28 |
| Higher | 2.61 | 0.38 |
| **Religion** |  |  |
| Hindu® |  |  |
| Muslim | 1.11 | 0.90 |
| Christian | 1.72 | 0.58 |
| Others | 1.12 | 0.89 |
| **Social group** |  |  |
| SC® |  |  |
| ST | 1.91 | 0.52 |
| OBC | 1.81 | 0.55 |
| Other | 1.67 | 0.60 |
| **Household wealth index** | |  |
| Poorest® |  |  |
| Poorer | 1.69 | 0.59 |
| Middle | 1.85 | 0.54 |
| Richer | 1.93 | 0.52 |
| Richest | 1.98 | 0.50 |
| **Region of residence** | |  |
| North® |  |  |
| Central | 1.99 | 0.50 |
| East | 1.84 | 0.54 |
| West | 1.37 | 0.73 |
| Southern | 1.58 | 0.63 |
| North-east | 2.17 | 0.46 |
| **Exposure to mass media** | |  |
| No exposure® |  |  |
| Low exposure | 2.07 | 0.48 |
| Medium exposure | 2.29 | 0.44 |
| High exposure | 1.30 | 0.77 |
| **Discussed menstrual hygiene with healthcare workers in past 3 months** | | |
| No® | 1.01 | 0.99 |
| Yes |  |  |
| **Working status** |  |  |
| Not Working® |  |  |
| Working | 1.12 | 0.89 |
| **Ownership of bank account** | |  |
| No® |  |  |
| Yes | 2.86 | 0.35 |
| **Ownership of mobile phone** | |  |
| No® |  |  |
| Yes | 1.51 | 0.66 |
| **Problem regarding getting medical help for self: getting permission to go** | | |
| No problem® |  |  |
| Big problem | 1.59 | 0.63 |
| Not a big problem | 1.41 | 0.71 |
| **Problem regarding getting medical help for self: getting money needed for treatment** | | |
| No problem® |  |  |
| Big problem | 2.15 | 0.47 |
| Not a big problem | 1.72 | 0.58 |
| **Problem regarding getting medical help for self: distance of health facility** | | |
| No problem**®** |  |  |
| Big problem | 3.25 | 0.31 |
| Not a big problem | 2.35 | 0.42 |
| **Problem regarding getting medical help for self: transportation** | | |
| No problem® |  |  |
| Big problem | 3.00 | 0.33 |
| Not a big problem | 2.22 | 0.45 |
| **Mean VIF** | **1.65** |  |
